# A Longitudinal Study of Memory Advantages in Bilinguals

**DOI:** 10.1371/journal.pone.0073029

**Published:** 2013-09-04

**Authors:** Jessica K. Ljungberg, Patrik Hansson, Pilar Andrés, Maria Josefsson, Lars-Göran Nilsson

**Affiliations:** 1 Department of Psychology, Umeå University, Umeå, Sweden; 2 School of Psychology, Cardiff University, Cardiff, United Kingdom; 3 Department of Psychology, University of the Balearic Islands, Palma, Spain; 4 Department of Psychology, Stockholm University, Stockholm, Sweden; 5 Department of Statistics, Umeå School of Business and Economics, Umeå University, Umeå, Sweden; Utrecht University, The Netherlands

## Abstract

Typically, studies of cognitive advantages in bilinguals have been conducted previously by using executive and inhibitory tasks (e.g. Simon task) and applying cross-sectional designs. This study longitudinally investigated bilingual advantages on episodic memory recall, verbal letter and categorical fluency during the trajectory of life. Monolingual and bilingual participants (*n = *178) between 35–70 years at baseline were drawn from the Betula Prospective Cohort Study of aging, memory, and health. Results showed that bilinguals outperformed monolinguals at the first testing session and across time both in episodic memory recall and in letter fluency. No interaction with age was found indicating that the rate of change across ages was similar for bilinguals and monolinguals. As predicted and in line with studies applying cross-sectional designs, no advantages associated with bilingualism were found in the categorical fluency task. The results are discussed in the light of successful aging.

## Introduction

The increasing number of older adults in western societies has led to the important question of how to improve quality of life in older age. In particular, research has recently focused on factors that can help to reduce the negative effects of aging on cognitive functions (e.g., [1], [2], [3], [4]) and that can potentiate what has been known as successful or optimal aging [5], [6]. For example, several studies have shown that physical exercise (e.g., [7]), education and engagement in mental activities after retirement (e.g., [8]) can play protective roles in cognitive aging.

There is now also evidence that the ability to speak two or more languages improves cognitive performance in certain tasks (e.g., [9], [10]), and that bilingualism may have some protective effect against dementia [11], [12], [13] by promoting some kind of cognitive reserve through life [14]. Comparing bilinguals and monolinguals on cognitive performance has shown that bilinguals outperform monolinguals in tasks such as the Simon task (e.g., [15], [16]), Flanker tasks (e.g., [17], [18]), the attention network task (ANT, [19]), the Stroop task (e.g., [20]), the letter fluency task [10] and card sorting tasks (e.g., [21]). This advantage is commonly explained by a relationship between managing two or more languages and enhanced executive control both for children and adults [9]. Bilinguals seem to rely on their ability to recruit the executive control systems in order to simultaneously activate and use their languages, a process that is concluded to depend on the continual practice in switching between languages and inhibiting unwanted information (e.g., [22], [23]). Benefits seem therefore to be closely connected to specific task features such as conflict, attentional control or switching (for comprehensive review see [10], [24]), and these processes are in turn concluded to rely heavily on brain networks related to prefrontal cortex, anterior cingulate cortex and caudate [10]. Studies also indicate differential brain activation patterns in bilinguals compared to monolinguals (e.g., [25], [26], [24], [10]), when exposed to tasks involving conflict and that retrieval processes in relation to lexical and semantic information has proven to be more effortful in bilinguals than in monolinguals. For example, the results from a PET-scanning study [27] suggested that bilinguals showed more demanding processing (more activation of cognitive control processes) in terms of increased activation in temporal structures and in left hemispheric inferior frontal brain areas when using the second language. Importantly, it has been shown that the difference in brain activation between the first and second language decreases as the proficiency in the second language increases, indicating less processing effort (e.g., [28], [10]).

However, there is still an important question that has not yet been systematically investigated, that is, the extent to which the enhanced executive control found in bilinguals may also benefit episodic memory (e.g., [10]); a function that starts to decline around late middle age (e.g., [29], [2]). It is likely that the bilingual advantage in terms of executive control may be extended to verbal episodic memory processes since this memory system is associated with activation in the prefrontal cortex (e.g., [30], [31]); similarly as memory processes that call upon attentional control or switching [10]. It is also generally agreed that memory retrieval relies on two different processes –recollection (remembering) and familiarity (knowing) – and that executive functions are related to recollection and not to familiarity [32]. Based on these assumptions, Wodniecka, Craik, Luo & Bialystok [33], studied the effect of bilingualism on verbal (familiarity and recollection of words) and non-verbal (familiarity and recollection of faces) memory performance in two experiments. The authors revealed small beneficial effects of bilingualism on recollection in the non-verbal memory tasks in the first experiment and in the verbal task in the second experiment. Both effects were found only in the most demanding conditions. The authors concluded that the bilingual advantages were closely connected to the retrieval processes involving higher demands on executive control.

On the same theme, Fernandes, Craik, Bialystok and Kreuger [34] investigated the performance (encoding and retrieval) of younger and older monolinguals and bilinguals in an episodic memory task with and without distraction (divided attention). Surprisingly, the authors found no advantages in performance associated with bilingualism. Instead, decreased performance on word recall was found in bilinguals compared to monolinguals. Smaller vocabularies in bilinguals were discussed as a possible explanation. However, except for these two studies there is at least one more recent study, which found support for a bilingual advantage in episodic recall [35].

Worth noting is that many bilingual studies have applied a cross-sectional design, using participants with different countries of origin, and a mixture of languages (e.g., [34], [36]; see also [37] & [38] for reviews), while no study (as far as we know) has been conducted using a longitudinal design with participants learning their second language mainly through formal education, sharing the same first language, the same culture and being tested on their first language. It might therefore be relevant for this research area to know whether memory benefits found in bilinguals are visible for anyone who can enter the formal educational system to learn and then frequently use a second language, but also if this advantage persists across life.

The general hypothesis of the current study is that learning and frequently using a second language will enhance executive/frontal processes (e.g., [9], [22], [23], [24]). Based on the bilingual advantages found in tasks involving executive/frontal processes, the aim of this study was to investigate whether performance in episodic memory recall tasks is facilitated in bilinguals compared to monolinguals and whether this advantage persists during the trajectory of life.

This study also included verbal fluency tasks (categorical and letter fluency). Based on earlier findings from cross-sectional studies (for an extensive review see [10]), our prediction was that bilinguals would show enhanced performance effects in verbal letter fluency across age, because of its closer connection to executive control systems and frontal lobe processing, Performance in a verbal categorical fluency task (semantic processing) on the other hand is strongly connected to vocabulary size (which can be a disadvantage for some bilinguals) and is related to activation in other brain areas such as the left inferior temporal cortex [39]. No beneficial effects due to bilingualism were therefore expected in verbal categorical fluency.

## Method

### Participants

The participants were drawn from the Betula Prospective Cohort Study of aging, memory, and health ([40], [41], www.betula.su.se). The study uses a stratified random sampling strategy with evidence of high population validity [40], [41]. Data has been collected at five test waves; 1988–90 (T1), 1993–95 (T2), 1998–2000 (T3), 2003–2005 (T4) and 2008–2010 (T5). At T1 participants were divided into age cohorts; 35, 40, 45, 50, 55, 60, 65, 70, 75 and 80. Each cohort consisted of 100 persons and the total number of trial participants (Sample 1) at the first test round was thus 1,000. At T2 (1993–95) 86% of test participants returned from S1 and a new sample was added; S3. The new sample (S3) was divided into age cohorts 40, 45, 50, 55, 60, 65, 70, 75, 80 and 85 (i.e. the same age as S1 participants at T2). All participants were screened for dementia; sensory impairments, mental retardation and a native tongue other than Swedish (see [40] for further details concerning recruitment and inclusion criteria). The Betula study was approved by the regional Medical Ethical Committee at Umeå University.

**Figure 1:**
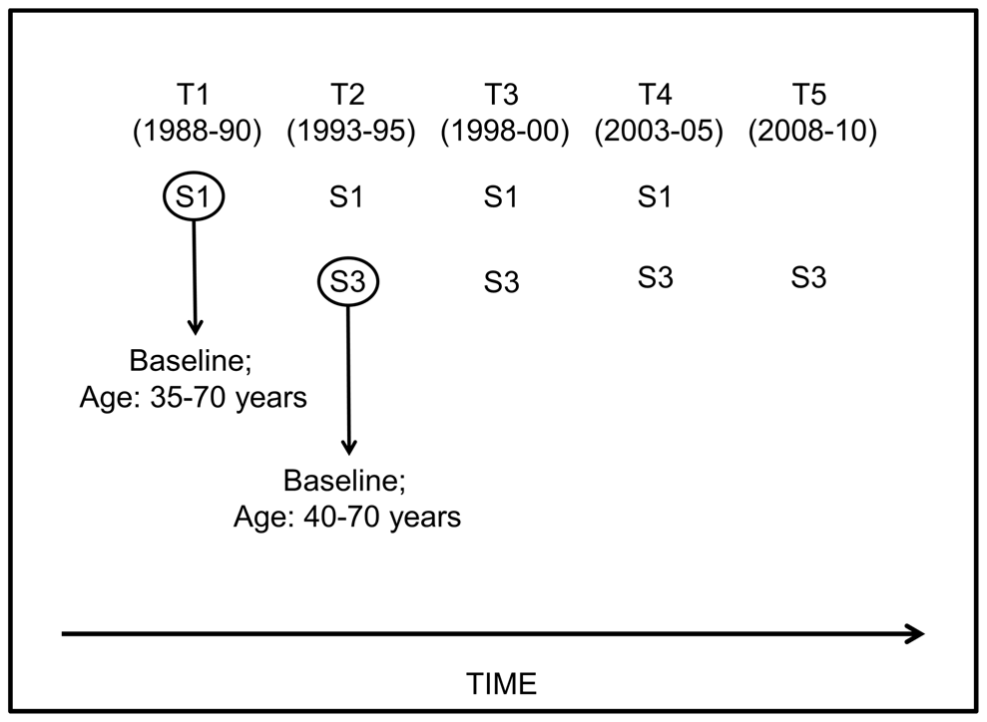
The design of the Betula study showing age at baseline and from which Samples (S) and Test wave (T) the data for current study originates.

For the present study, a total sample of 178 participants (44.9% women) from four test waves (T1 to T4 for S1 and T2 to T5 for S3) was used. The age at baseline (i.e., T1 or T2) ranged from 35 to 70 years (*M* = 49.9, *SD* = 9.9), see Figure 1. Based on a self-reported question regarding ability to speak a second language, those who did, completed a self-reported “Language History Questionnaire” ranging from 1 (very poor) to 6 (excellent). Measures of ability to read, write, speak and listen to a second language were collected and participants with a score of 4 and higher across all abilities were categorized as bilinguals, while participants using only one language (i.e., Swedish) were categorized as monolinguals. The majority (95%) of the bilinguals in this study reported English as their second language; they began to learn English in primary school (at the age of 9), and had approximately 7 years of formal training. 64% of the participants indicated that they mainly used their second language “when traveling”, 29% “at work”, and 7% “at home”. Approximately 80% of the bilingual participants indicated that they spent between 0 and 2 hours a day reading, writing, speaking, and listening in their second language. All participants were native Swedish speakers living in Sweden and all tests were in Swedish. Table 1 provides demographic data for monolingual and bilingual participants.

**Table 1:** Summary of Sample Characteristics.

	Monolinguals	Bilinguals
Variable		
Age		
Range	35–70	35–70
* M*	54.8	46.5
* SD*	9.3	8.9
Years of education		
Range	9–17	9–17
* M*	10.7	14.3
* SD*	2.0	2.1
Gender (female)	27%	53%
* N*	74	104

Note: *M* = mean, *SD* = Standard Deviation.

### Procedure

During the waves of assessments, collections of measures from two sessions were conducted during each test wave. Each test session lasted around 1.5–2 hours for each participant, in which the first included a health examination and questionnaires, and the second comprised an extensive battery of cognitive measurements. All participants signed a written informed consent and during the test session participants were requested to use glasses or hearing aids if normally used, and they were all tested individually.

#### Episodic memory: recall

This construct reflects a composite score, based on unweighted raw-scores, from three different recall tasks. *Recall of actions and sentences:* Participants were presented with two lists of 16 verb-noun sentences, each denoting a simple action (e.g., lift the book). For one list, participants were requested to enact each sentence, using the specified object (8 seconds/item). The other list was studied without enactment. A free recall test of the sentences followed after each list (enacted/actions and without enactment/sentences). The number of correctly recalled sentences from the two lists was used as a measure of performance. *Category cued recall of nouns:* Following the free-recall test, participants were provided with a sheet listing the eight semantic categories into which the nouns of the 32 sentences (described above) could be divided. They were told that these might serve as cues to remember the nouns. Number of nouns recalled from enacted/actions and without enactment/sentences served as a measure in the analyses. *Recall focused attention:* Participants were presented with a list including 12 words. The items in the list were read aloud by the experimenter at a pace of 2 seconds/item. Following presentation of the last item of the list, participants recalled as many of the words as possible in any order at a given pace (2 seconds/item), indicated by a metronome. The number of correctly recalled words was used as a measure of performance [6], [42].

#### Letter fluency

The participants were required to generate as many words as possible with an initial letter A during one minute [42].

#### Category fluency

The participants were required to generate as many occupations as possible with an initial letter B during one minute [42].

#### Visuospatial ability

The WAIS-R Block Design test was used [43], [42]. Its inclusion is motivated by the fact that this test is usually considered a good measure of general fluid ability, *Gf*, [43], [44]. In the current study the block design test is used as a covariate, controlling for general fluid ability at baseline.

### Statistical Analysis

To be able to compare the performance of bilinguals and monolinguals in the cognitive tasks as a function of age, random coefficient mixed model analyses were applied [45]. Separate analyses (statistical models) were conducted for each of the three cognitive measures (episodic memory recall, letter fluency and categorical fluency). These models enabled the estimates of the mean path of change in performance, modeled as fixed effects, in each of the three cognitive measures and characterized how individual paths differed from the mean trajectory, modeled as random effects. A practical advantage of this approach is that it allows for individuals not having the same number of observations and/or varying initial ages [45] to be included in the sample.

Number of correct responses in the cognitive tasks was modeled by using a dummy variable (Bilingual) indicating if the participant was bilingual or not, coded 1 for bilinguals, and 0 for monolinguals. This dummy variable was modeled both as a fixed main effect but also as an interaction with age and age squared as predictors.

These models explored whether cognitive performance differed between bilinguals and monolinguals, in number of correct responses, and if there was an interaction between the two language groups and age/age squared. Analyzing possible interactions between groups (bilingual versus monolingual) with age was aimed at revealing whether there were differences in change as a function of age (linear age effect), and the quadratic trend (age squared) allowed analyses of possible accelerations of cognitive decline among the oldest participants. To rule out potential confounds in assessing these differences, the following variables were included as fixed effects and in interaction with age and age squared: education (in years), gender (indicator variable coded: 1 for female, 0 for male) and general fluid ability (Gf) at baseline. To ease interpretation of regression coefficients and reduce multicollinearity [46], continuous variables were centered by subtracting the round number closest to the respective variable’s mean; age at 50, education at 13 years, and Gf score at 31. When variables are centered, the interpretation of the main effect of bilingualism on performance in the cognitive tasks is characterized by a reference person, i.e. a 50-year old male participant with 13 years of education and a Gf score of 31 (a male participant whose age, years of education and Gf score are equal to the average in the sample).

The models also included subject-specific random effects for both intercept and linear age effect across 15 years. To reduce skewness, which may yield biased parameter estimators, the variables Letter Fluency and Categorical Fluency were log-transformed.

A control for a dropout effect was made, by including a dummy variable indicating if the participant dropped out between T4 and T5 (N = 33), which was found non-significant, and hence excluded from the models. All analyses were performed using the lmer module of the lme4 package, R software, Version 2.14.2 [47], [48], p-values were computed with Markov chain Monte Carlo sampling (using the pvals.fcn module of the languageR package).

## Results

For the total sample, number of correct responses at baseline in the Episodic memory task ranged from 15 to 64 (*M* = 38.4, *SD* = 9.4), in the Letter Fluency task they ranged from 3 to 27 (*M* = 13.0, *SD* = 5.0, and in the Categorical Fluency task they ranged from 0 to 12 (*M* = 5.1, *SD* = 2.4).Years of education ranged from 9 to 21 (*M* = 13.0, *SD* = 2.9), and number of correct responses in the Block Design test (general fluid ability) at baseline ranged from 4 to 51 (*M* = 30.7, *SD* = 9.5). Table 2 shows descriptive statistics from baseline (first) measurements in more detail for each age cohort and language group respectively.

**Table 2:** Descriptive statistics including mean (*M*) and standard deviation (*SD*) are shown for each dependent measure across age cohorts and language groups at baseline.

	Measures (at baseline) and *N*’s								
	Episodic Recall		Letter Fluency		Category Fluency		General Fluid Ability		*N*
Group/Age Cohort	*M*	*SD*	*M*	*SD*	*M*	*SD*	*M*	*SD*	
Monolinguals									
35	33.67	13.50	9.33	4.04	3.33	0.58	22.33	1.53	3
40	29.33	7.42	9.50	3.39	4.17	2.32	20.67	12.21	6
45	31.00	5.76	8.83	2.99	3.33	2.50	28.50	14.34	6
50	34.46	8.66	11.85	2.54	5.69	2.02	30.69	9.79	13
55	36.69	8.30	8.94	4.20	4.81	1.91	30.31	6.68	16
60	33.13	7.54	10.19	3.83	4.31	2.36	27.00	6.40	16
65	27.00	8.29	9.50	5.24	3.17	0.75	18.00	9.19	6
70	32.38	7.03	10.38	3.02	5.25	2.49	22.88	7.66	8
Bilinguals									
35	43.80	6.49	14.70	6.15	4.90	2.64	32.60	8.53	10
40	44.85	8.18	14.80	4.21	5.76	2.54	35.06	7.61	34
45	40.74	8.23	15.91	4.36	5.74	2.49	34.48	8.83	23
50	41.19	8.34	15.75	5.31	5.63	2.42	33.94	5.66	16
55	40.17	11.13	13.00	6.48	4.17	2.14	31.50	14.07	6
60	41.83	5.85	18.17	4.02	5.00	1.26	31.00	8.65	6
65	37.17	11.70	14.83	5.23	5.67	3.20	28,33	12.18	6
70	36.67	8.50	15.33	5.77	5.00	1.73	31.00	13.11	3

**Figure 2:**
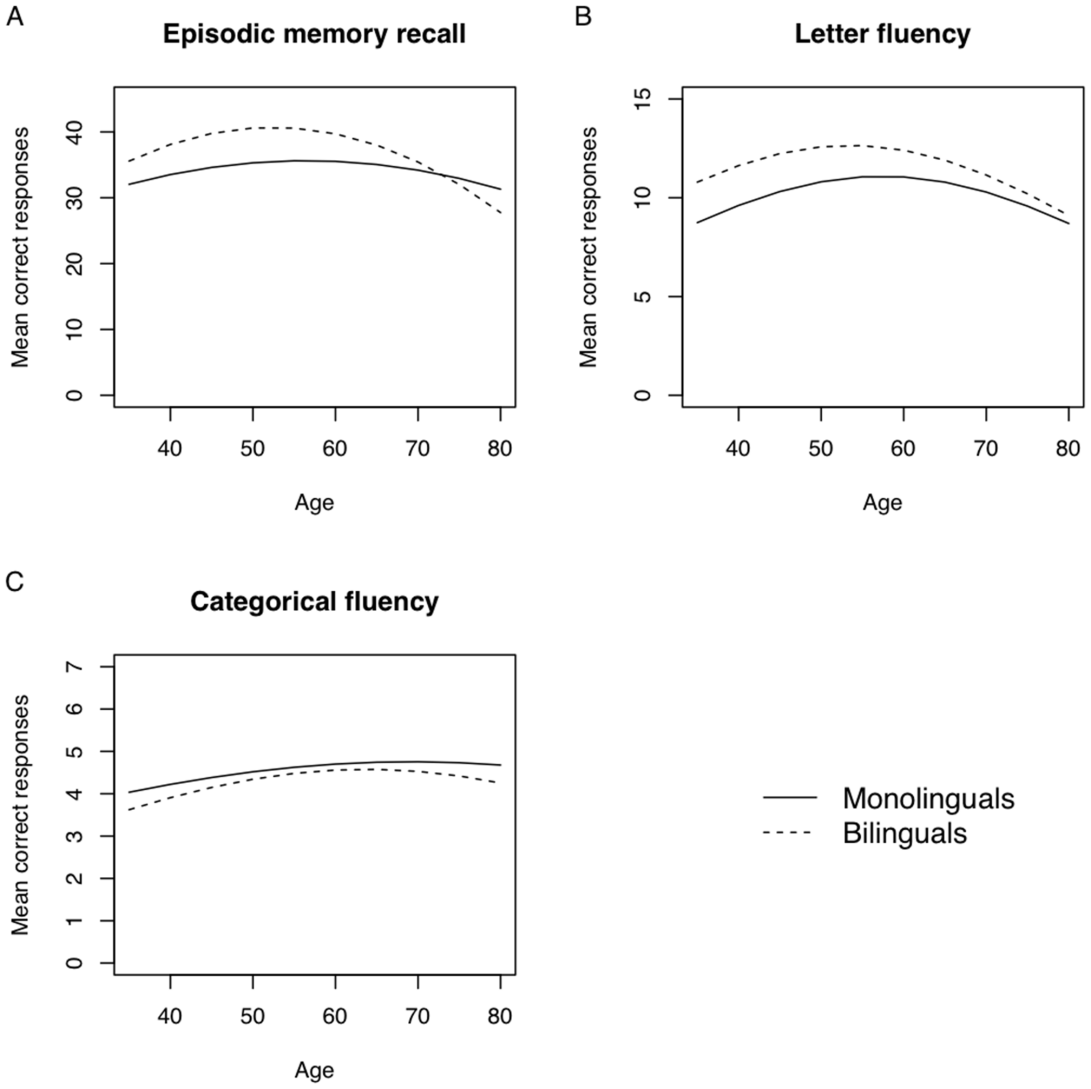
Fitted (longitudinal) average memory scores, for Episodic memory recall (Panel A), Letter Fluency (Panel B) and Categorical fluency (Panel C), as a function of chronological age, for males with average values for: education (13 years) and General fluid ability (Gf = 31), based on estimates from Random effects model: bilinguals (dashed line) monolinguals (solid line).

Results from the random-effect models are displayed in Table 3. Figure 2 shows an overview of the fitted longitudinal average (for the reference person) in number of correct responses in the three cognitive tasks across all age groups.

**Table 3:** Parameter estimates and standard errors (*SE*) from the random effect models for the three cognitive domains.

	Analysis		
Model Parameter	Episodic memory recall	Letter fluency	Category fluency
Fixed effects			
ntercept	35.308 (1.134)**	2.469 (0.043)**	1.708 (0.056)**
Bilingual	5.294 (1.519)**	0.140 (0.057)**	−0.032 (0.074)
Education	0.928 (0.237)**	0.051 (0.009)**	0.032 (0.011)**
Female	3.494 (1.089)**	0.104 (0.041)**	0.085 (0.052)*
Gf	0.193 (0.062)**	0.008 (0.002)**	0.010 (0.003)**
Age	0.100 (0.098)	0.006 (0.004)	0.004 (0.006)
Age squared	−0.008 (0.004)*	−0.004 (0.000)**	−0.000 (0.000)
Bilingual × Age	−0.020 (0.124)	−0.003 (0.005)	0.002 (0.007)
Bilingual × Age squared	−0.009 (0.006)	−0.000 (0.000)	−0.000 (0.000)
Education × Age	−0.012 (0.017)	0.001 (0.001)*	0.000 (0.001)
Education × Age squared	−0.000 (0.004)	0.000 (0.000)*	0.000 (0.000)
Female × Age	−0.033 (0.083)	−0.006 (0.003)	−0.006 (0.005)
Female × Age squared	−0.001 (0.038)	0.000 (0.000)*	−0.000 (0.000)
Gf × Age	−0.000 (0.005)	0.000 (0.000)	−0.000 (0.000)
Gf × Age squared	−0.000 (0.000)	0.000 (0.000)	0.000 (0.000)
Variance components, Random effects			
Intercept	34.172 (5.846)	0.044 (0.210)	0.054 (0.231)
Slope	0.026 (0.162)	0.0001 (0.007)	0.0004 (0.006)
Residual	23.124 (4.809)	0.041 (0.202)	0.109 (0.330)

*Note:* The table lists restricted maximum likelihood estimates (REML). Standard errors are shown in parentheses. Bilingual were dummy-coded such that bilinguals was compared with monolinguals (bilingual = 1, monolingual = 0). Gf = General fluid ability. All predictors are centered around the variable mean, such that that primary coefficients, Bilingual, and Bilingual in interaction with Age and Age squared are compared for a reference person i.e. Age = 50 years old, Gender = Male, Education = 13 years and Gf = 31. The interpretation of the fixed effects shown is equivalent to that of parameter estimates in ordinary least squares regression. The models also included random effects for intercept, linear rate of change (Slope), and residual.

* *p*<.05,

** *p*<.01.

### Episodic Memory

In the first analysis, we modeled the repeated measures of correct responses in the episodic memory task as response using a random-effects model.

The intercept showed the expected number of correct responses in the episodic memory task for a reference person among monolinguals (*b = *35.31, *SE = *1.13, *p*<.001), and evidence of an accelerating age-based memory decline (linear age trend *b* = 0.10, *SE = *1.52, *p = *.070; quadratic age trend *b* = −0.01, *SE = *0.01, *p = *.031) across all age groups.

As indicated by the term Bilingual in Table 3, bilinguals performed in general better in the episodic memory task already from baseline, showing on average 5.29 units better performance than monolinguals in correct responses (*SE = *1.52, *p*<.001), compared to the monolingual group. Moreover, no significant interaction was found, indicating that the age-based rate of change did not differ significantly between monolinguals and bilinguals (see Figure 2).

### Letter and Categorical Fluency

An analysis of the results of performance in the Letter fluency task showed that for a reference person, monolinguals had an average of correct responses of 2.47 (*SE = *0.05, *p*<.001), and a significant age-based change (linear age trend: *b = *0.01, *SE = *0.00; *p = *.110; quadratic age trend: *b* = −0.00, *SE = *0.00, *p = *.020). A bilingual advantage was detected in terms of significantly better performance in bilinguals compared to monolinguals, *b = *0.14, *SE = *0.04, *p*<.001), but no significant differences due to an age-based change between the groups were revealed (linear age trend: *b* = −0.00, *SE* = 0.02, *p* = 0.51; quadratic age trend: *b* = −0.00, *SE* = 0.00, *p = *.92).

No significant differences in performance were found between monolinguals and bilinguals in the Categorical Fluency task.

## Discussion

The objective of this study was to explore the longitudinal relationship between bilingualism and performance in episodic memory recall, letter fluency and categorical fluency. Better performance in episodic recall and letter fluency was expected for bilinguals compared to monolinguals across the life span. No differences were, however, expected in the categorical fluency task, in accordance with earlier cross-sectional findings (e.g., [49]). By using a well-established statistical Random effects model allowing individuals not having the same number of observations and/or varying initial ages [45] and despite controlling for education, gender, and general fluid ability, the results from this longitudinal model showed a bilingual advantage in the performance of verbal episodic recall, and this benefit persisted across age. No interaction effect between the performance of the bilinguals and monolinguals and age was found indicating that during this period of life (35–85 years), bilinguals outperformed monolinguals in this type of task to the same extent across all ages.

Morton and Carlson [38] (see also [50]) have recently argued that it is possible that the bilingual advantage on executive functions observed in previous studies (see [9] for a review) is linked to cultural factors. As Paap and Greenberg [50] claim, matching language-groups on factors that influence the development of executive functions is a serious challenge, and particularly difficult when the bilingual group is either from a different culture or bicultural (p. 256). It is therefore possible that the bilingual advantage observed is explained by factors that have more to do with the cultural factors than with the bilingualism per se. It is important to mention that this hypothesis does not apply to the current study, however. Both our monolingual and bilingual participants were Swedish nationals and shared the same culture from birth.

The present study extends previous findings [34], [33] by showing that bilinguals outperform monolinguals in verbal episodic recall across age when applying a longitudinal, repeated measures design. Importantly, the present study differs from earlier studies with respect to the compositions of the sample groups. All participants in the present study shared the same first language (Swedish) and all were tested in Swedish. This choice of sample helps bolster conclusions by eliminating common confounding factors such as vocabulary size when comparing monolinguals and bilinguals (e.g., [10], [49]). The results from this study may also imply that the bilingual advantages in certain memory functions are extended to include bilingual groups that are not as frequently exposed to the second language as were the bilingual participants included in earlier studies (i.e. the use of immigrants, [10]). Based on these findings, we conclude that living in a society (or moving countries) in which the second language has to be used daily in the society etc. is not a necessary condition to observe bilingual advantages.

The ongoing discussion about cognitive reserve [14] is an important topic for the understanding of for example successful aging. This study adds another piece to the puzzle by showing that learning and using a second language to a (subjectively) fluent level might optimize memory performance across age. A similar conclusion has been reached by other studies focusing on dementia [11], [12], [13].

The quadratic shape of the curves displayed in Figure 2, might at first seem odd. However, this is due to a well-established test-retest effect because of the longitudinal design of the Betula study [51]. Since this effect affects all participants to a similar degree it has no impact on the group differences. The similar rate of memory decline across age in the two groups may also come as a surprise if one expects age to interact with bilingualism showing an age-related decline in episodic memory reduced in bilinguals compared to monolinguals. On the contrary, having a look at the estimated performance for bilinguals and monolinguals in episodic memory (Figure 2), despite the lack of statistical significance, performance in the bilingual group obviously drops later in life (visually around 65-years), ending up on a similar performance level as in the monolinguals. It is important to note that there is a growing body of evidence indicating that the preservation of cognitive functions may not be a matter of building up a cognitive reserve during younger years to be used later in life. Instead, a constant activation in terms of mental and socially stimulating activities might prevent a negative change in cognitive functions in older age (for an extensive review, see [2]). This might suggest that the bilinguals in the current study were benefiting from the advantages provided by their bilingualism as long as it was regularly used during their professional life. For example, if they used their second language mostly at work (in this study 29% indicated that they did) and during a time when they were socially active (64% indicated using their second language mostly when traveling), this advantage might start to drop after retirement when most commonly individuals live a less active life. Additionally, as suggested by other longitudinal findings, during the same period of life, both episodic and fluency memory start to drop [29], [51], [52]. Based on the language questionnaire used in this study, 80% of our bilinguals indicated that they spent less than 2-hours a day reading, listening, talking and writing in their second language. However, this should be interpreted as a rather rough measurement as using a second language just below two hours per day may be considered as relatively high frequency use, whereas, say 20 min of use per day may be considered as relatively low frequency use. Contrary to the bilingual participants included in other studies [10], our bilingual participants were not able to practice their second language during their daily routines (e.g. when going to the supermarket, visiting the dentist etc.). What is important however is that the use of the second language investigated in this study was sufficient to improve and maintain a higher memory and letter fluency performance across age.

The visual drop of memory performance in the mid 60′s mirrors a similar phenomenon that has been shown by two other longitudinal studies in which complexity of occupation was proven successful to gain and maintain a higher cognitive performance during main life-time occupation [53], [54]. However, in both studies the authors found a sharp drop in performance in the groups with high complexity occupations compared to those with low complex or low routine jobs after retirement. Similar conclusions were drawn in a review about cognitive training by Salthouse [55].

Another interesting finding of this study was the enhanced effect on letter fluency in the bilingual group. It is well established that letter fluency requires more executive processing than categorical/semantic fluency. For example, Martin et al. [56] tested normal participants in dual task conditions and found that a concomitant finger-tapping task (putatively, a frontal lobe task) disrupted letter fluency, while an object decision task (putatively, a temporal lobe task) disrupted category fluency. Also, Baldo et al. [57] have shown that letter and category fluency deficits correlate with lesions in frontal and temporal cortices, respectively. Finally, neuroimaging studies have revealed that performance in letter fluency tasks seem to be related to grey matter density in a medial frontal region, whereas performance in categorical fluency is related to increased grey matter density in left inferior temporal cortex [39]. Although the categorical fluency task used in the Betula project also involves a phonemic aspect (occupations starting with B), we argue that this task, relatively speaking, is more heavily weighted toward semantic retrieval. In our study, bilinguals outperformed monolinguals as predicted, while no differences were found in the categorical fluency task. The bilinguals’ advantage in the letter fluency task is known from cross-sectional studies in which it is concluded that bilinguals benefit from this task because of its higher demands on executive control (e.g., [49]). However, no differences between groups are typically found in verbal categorical fluency, because of its connection to more semantic processes.

In sum, a bilingual advantage has for the first time been demonstrated in verbal episodic recall, applying a longitudinal method, using test samples sharing the same first language. Better performance on verbal letter fluency was also observed when bilinguals outperformed monolinguals during the trajectory of life (a period of approximately 45 years). With respect to the cognitive reserve hypothesis [14], these findings show a clear advantage during a period of time in which most of the participants in this study lived an active life (before retirement). However, based on Nyberg et al.’s [2] conclusions; that a beneficial memory effect may depend on the degree of mental and social activation; it is unclear how long this positive effect will persist. This advantage may decline after retirement (in Sweden around the age of 67) when people live a less active life, and thereby reducing the use of their second language.
